# Professional Ollapodrida

**Published:** 1859-09

**Authors:** 


ATLANTA

### Medical and Surgical Journal.

Vol. V.] SEPTEMBER, 1859. [No. I.

### ORIGINAL COMMUNICATIONS.

### ARTICLE I.

*Professional Ollapodrida.*—By a Retired Pharmacien.

No. 2.

*Nitrate Potassa* is much more extensively adulterated now than formerly, with common salt; it is of course easily detected by a little attention, without resorting to the conclusive test, the nitrate of silver. Adulterated samples are generally found in small fragments.

This salt was a favorite ingredient in cough mixtures of Dr. Whittaker, of New York. ℞—Powdered Acacia, 3 drachms; Nitrate Potassa, 30 grains; Tartar Emetic, 2 grains; Syrup Tolu, 1 oz.; Paregoric, 4 drachms; water, 6½ oz—mix. Dose: tablespoonful.

*Citric Acid.*—In portions of Florida, extensive groves of sour oranges exist, from which almost any quantity of fruit may be obtained in its season. Query: might not the juice of this fruit be made a profitable source of citric acid? The fruit could be crushed and pressed in the ordinary cider apparatus, and neutralized with chalk. The crude citrate of lime, which precipitates when separated and dried, is in a fit state for transportation to the manufacturing chemist.

*Chloroform.*—The writer has but little experience with the administration of chloroform in labor; is satisfied, however, that it does in some instances undoubtedly control uterine contractions; and in the second or expulsive stage, endanger the life of the child, and perhaps subsequent inflammation and sloughing of the maternal tissues. Would consider it good practice, if the chloroform be given, to have a good pair of reliable forceps at hand, ready to command the labor, in case of suspension of the uterine efforts; and still better practice, never to make use of a drug to convert a normal labor into an abnormal one.

For the external application of chloroform the following formula is a good one. ℞—Chloroform; Tinct. Opium; Tinct. Camphor; of each 1 oz.; Camphorated Tinct. of Soap, 3 oz.—mix, and direct to be applied upon a piece of flannel, and covered with oil silk or a folded towel. This liniment is an excellent internal remedy for colic in the horse, given in the dose of an ounce, and repeated as indicated.

℞—Chloroform, 1 oz.; Fluid Extract Valerian, 1 oz.; Camphor Water, 2 oz.—mix, and drink a half teaspoonful dose. A favorite remedy in some forms of neuralgia, as also colic, and the acute pains of dysmenorrhœa.

*Lactucarium* is useful in the case of certain nervous, hysterical women who do not bear opiates well: ℞—Lactucarium, Lupuline and Camphor, equal parts, mix and make into 5 grain pills, of which 1 or 2 may be a dose. Generally no moistening material is required in making this pill, although separately dry, yet the materials when incorporated thoroughly in the mortar (which should be warmed in cold weather,) become so adhesive as to readily be formed into pills.

*Hydrargyrum Cum Creta*, as usually found in the shops, is for the most part unsatisfactory to the physician; that which is manufactured upon the large scale often vomits children, perhaps from the presence of black oxide of mercury; while as usually made by hand, the mercury is not fully extinguished, and therefore is not absorbed. The powdered blue pill, prepared as suggested by Edward Parrish, would probably be a more efficient remedy, and might be combined with chalk if desired. Parrish prepares the powder by rubbing the materials in a mortar, substituting powdered roses and sugar for the conserve ; the extinguishment of the mercury is accomplished much more speedily in the dry way than by the old method. The Baltimore College recommends that the blue pill be washed with water until the honey is removed, dried, and so much of a mixture of sugar of milk and starch be added as will make up the loss in weight. This powder is not only a good substitute for the cretaceous preparation, but is often preferred by patients to the pill, and may readily be converted into the latter form when desired.

The writer has seen children taking the H. cum creta day after day, without effect, satisfactorily impressed with two or three small doses of the mild chloride. An infant, dangerously ill, had the former in large doses, and continued until the numerous metalic globules were distinctly visible upon the guard napkin, all to no purpose. It was believed that the life of the child was saved by a few doses of the more reliable sub-chloride. We may temporize with the creta, but when we want to *do something*, the other is the remedy.

*Calomel.*—Formerly the hydro-sublimed calomel was prepared only by some of the English manufacturing chemists, and was justly preferred to the American article. Now, Powers & Weightman, of Philadelphia, manufacture a hydro-sublimed calomel equal to any the chemical world affords. Very few of the country orders for "English calomel" are ever filled with that article of prime quality. Too often is it the case that such orders bring an article of American manufacture *inferior* to that of P. & W. The English, when genuine, is probably in no wise superior, and, in consequence of the tariff, is considerably more expensive. It is usually put up in two pound bottles.

*Citrine Ointment* is a preparation which requires much skill and care to make always of standard quality. Very few of the samples in market, if right at first, will stand the test of time. The acid must be of proper strength and heat applied just right to secure a good result, and it is believed that the substitution of lard oil for the neatsfoot oil and lard, affords a more uniform and handsome preparation.

*Iodine Preparations.*—The small portion of iodide potassium added to the formula for the ointment at the last revision of the Pharmacopœia, plays an important part in effecting the solution of the iodine in its perfect distribution through the mass of lard. It should never be omitted.

The tincture is not a fit preparation for internal use, unless iodide of potassium be added in sufficient quantity to insure the solubility of the iodine in water. It is believed that the addition of two drachms and a half of the iodide to each pint would greatly improve this tincture for all purposes to which it is applied as a remedy, and also fit it for internal use.

Nearly everybody knows the composition of the "Twiggs' Hair Dye," as it was called. ℞—Precipitated sulphur, 1 drachm; acetate lead, ½ drachm; rose water, 4 ounces—mix. This is doubtless essentially the "Prof. Wood's Hair Restorative," so much in vogue just now all over the country.

*Itch Ointment.*—℞—Brown soap 1 oz., salt ½ oz., sulphur ½ oz., alcohol 1 drachm, vinegar 2 drachms, chloride lime ½ drachm—mix. Apply thoroughly to the affected parts on going to bed at night. The mixture should be prepared for each case, as it will not keep long. Its efficacy has been well tested by the writer, as well as by others.

*Colchicum.*—The English wine from the fresh root, although expensive, is usually so much more reliable than any other preparation, as to fully warrant the additional cost.

*Sassafras.*—It is said that a little oil of sassafras added to the tincture of opium so far modifies its action upon the brain and nervous system generally as to do away with the necessity for black drop and the "Elixir." The experience of the writer in the matter is favorable, though entirely too limited to warrant any positive assertions.

*Ipecac.*—The active principle of the root rests almost entirely in the bark, which, in the grinding process, passes first into powder, and may be separated easily from the tougher ligneous fibre. Such powder is sold as "*Cortex Sine Ligno*," and although rather more expensive than the ordinary, yet it is equally cheap, in the long run, and altogether more satisfactory.

In making the syrup, it is desirable that the root be freshly ground for the purpose—at first very coarsely, to separate completely the ligneous portion, afterwards to a state of fine meal, from which the proper quantity is to be weighed. The tincture is procured by properly conducted displacement, evaporated upon a water-bath, to get rid of the alcohol, filtered to remove inert vegetable matter before adding the water to make up the measure. In the subsequent solution of the sugar, any long boiling is to be avoided—it would but injure the syrup, without any compensating advantage. The writer is in the habit of using two pounds avoirdupois of sugar, a small fraction under the two and a half pounds troy. The former weight is more nearly correct when the water is not entirely pure, and is much more conveniently attained with the ordinary scales.

This syrup is prone to slow crystalization, and this more particularly, if a little alcohol be left in evaporating the tincture. Very large and regular crystals of the sugar are often obtained from it. If decolorized by bone black, and very small portions of alcohol be added to the syrup from time to time, while the bottle is allowed to remain entirely at rest, it is probably the most reliable source of sample crystals for the cabinet. They may be obtained occasionally in single specimens of two or perhaps four ounces weight. When removed, the crystals should be washed off with high proof alcohol, and not by water, which would impair the beauty and polish of the planes.

If ipecac in fine meal be exhausted by dilute alcohol and the tincture concentrated, filtered, and further concentrated to a syrupy consistence in the water-bath, and incorporated with sugar, the half or entire weight of the ipecac used, it affords an elegant substitute for several forms of the drug, and is easily administered to children. Many of the crude vegetable remedies might be treated in the same way with advantage.

By the by, ipecac as an emetic, though a staple remedy with many, is not perhaps so frequently resorted to in general practice as its safety and superior efficacy would warrant. It is getting to be in too many instances considered *unrefined* to vomit, and even rather indelicate or perhaps brutal to propose such a measure, hence we purge over often and over much, and administer unnecessary quantities of mercurials. Now a proper delicacy and refinement in both practitioner and patient, is in the highest degree commendable, and rather to be encouraged as part and parcel of the true progressive spirit of the age, nor should the physician fail to give due weight to the tastes and inclinations of his patient, so far as he can consistently with his duty, to regard the substantial welfare of the latter. Away with the old idea of meeting, *ex necessitate*, the preferences of the sufferer with the thing he shall most *loath*, nevertheless it must often be felt and acknowledged that the failure to empty the stomach promptly of crude indigestible matter, or the more frequent failure to disgorge mechanically the liver and gall bladder, cannot be satisfactorily compensated by the use of the mercurial or other purgatives. In such cases it is fit that duty should overcome the scruples of refinement or childishness.

*Venesection* gives rise occasionally to warm discussion in the medical world, we have for the most part, now a days two classes of doctors; one feels his patients pulse and asks himself the question "*can* I bleed?" the other asks "*must* I bleed?" To the latter class belongeth this deponent.

*Tetter wash*, for ringworm and tetter upon the scalp or face of man particularly. ℞—Tinct. sanguinaria, acetic acid equal parts, mix and direct the affected parts moistened with it once daily.

*Tartarized antimony*, is one of those active drugs which it is highly desirable should be pure in quality, and uniform as possible in its action, as generally sold in powder it is often adulterated and for this reason is to be prefered in distinct well formed crystals, so easily is it reduced to powder in a mortar, there can be no necessity for powdering it upon a large scale, whereby an opening is made for easy adulteration.

*Antimonial ointment* is active in proportion to the purity and fineness of the powder, a superior article is made by dissolving the antimonial salt in water and precipitating by alcohol, the powder is very minutely divided, let it be dried, weighed and made into ointment, with lard, in winter and simple ointment in summer.

*Laxatives*—habitual constipation is an evil for the remedy of which every physician receives frequent call, modifications of diet with the use of laxative bread, often prove effectual and better than drugs, *habit* also is a powerful auxiliary which should not be overlooked, as also the "punchinello practice" of kneading the abdomen. Among the simpler remedies, a good one is: ℞—Powdered charcoal 1 ounce, carb. magnesia ½ ounce, powdered Ginger ½ ounce, mix and direct a teaspoonful 3 times daily—a teaspoonful of epsom salt taken every morning on getting out of bed, until the habit of daily stool is fully established, is usually quite satisfactory.

*Magnesia.*—The common calcined magnesia of the shops is hardly fit for administration. Henry's English preparation is usually esteemed the best, but is not perceptibly superior to Husband's American article, which costs but half the price, and Ellis's is perhaps equal to either.—Murray's fluid magnesia is merely a solution of a portion of carbonate in carbonic acid water.

*Castor Oil* of western manufacture frequently reaches us largely adulterated with lard oil, the east India article imported in cases of two-square ten gallon cans each, is usually very reliable. If the oil be rubbed up with a little calcined magnesia and flavored with a few drops of essence of mint, it is taken with more ease and comfort by all save infants.

*Extract Cannabis Indica* is attracting more notice now than formerly. It is offered in the market from several different manufacturers, differing most widely in appearance, as also in medicinal activity. Morson's English Extract is in color a dark walnut brown, of soft, pillular consistence; Tilden's American preparation, quite dark, almost black, consistency pretty firm; Herring's English is of a grass green color in thin stratum, a soft pillular mass; the extract of Squire (apothecary to Her Majesty Queen Victoria,) is peculiarly rich and showy, of a brilliant semi-transparent, rather grass-green hue, difficult to describe—rub up a little Prussian blue with white lead and compare the color with ultramarine, and you have an idea of the contrast between Herring's extract and Squire's —the consistence of the latter is soft and tarry. The odor of Squire's is strong and highly characteristic; Herring's next and Tilden's least of all.

The writer administered Morson's extract to a delicate, hysterical lady in three-grain doses, at intervals of two or three hours, without any manifest impression. In another attack, gave ½ grain Squires' and was called soon after to quiet the alarm of the patient and friends. She complained of prostration, with very peculiar and unpleasant sensations, which she could not describe. Her pulse had fallen from 80 down to 50, and continued so for some three hours, when it gradually rose to the normal standard. She was afterwards induced to try a quarter grain, with like results but in less degree. Another took a quarter grain, and in two hours another like dose. She was seized with a violent paroxysm of alternate laughter and weeping, such as had never occurred to her previously, nor since, and could not be induced to repeat the experiment.

Other patients have tolerated the smaller doses very well, but in no case, so far, with any signal advantage. No marked effect from the Morson's extract has been witnessed. Herring's and Tilden's were not experimented with. The former is probably inferior to Squire's, and better than Morson's; Tilden's probably least reliable of the four.

*Blisters.*—The application of mustard with a view to hasten and insure the subsequent vesication of a blister signally defeats the desired object. In spreading blisters, it is a good plan to have at hand an assortment of patterns of all desired shapes and sizes, cut in thin pasteboard and varnished. The pattern is placed upon the cloth, blister spread with a spatula, pattern lifted off brings with it all superfluity of cerate and leaves a plaster with clean, well-defined margin, looking ship-shape, as it should do. A desirable material, though an expensive one, upon which to spread the cerate is adhesive plaster cloth. This expense may be somewhat lessened by using bleached drilling. Take the inner piece of the pasteboard left in cutting the pattern—lay it upon the drilling and spread a narrow strip around it with adhesive plaster lump. By preparing an assortment of such cloths, and having them at hand, a handsome blister may be spread at once with ease and trifling expense. If the blister is to be sent out to the patient, it is well to sprinkle good powdered cantharides over the surface to prevent adhesion. The practice of dusting a little morphine upon the blister to prevent strangury in certain cases, is in general repute and usually quite successful.

*Burgundy Pitch,* as frequently, perhaps generally found in the shops, is rosin melted and incorporated with water. It strongly resembles the Canada pitch. but is very unlike true Burgundy.

*Nitrate Sil[illegible]* in stick is usually made of two qualities styled pure and No. 2; the latter containing a variable per centage of nitrate potassa or soda, according to the demands of different parties; the adulteration occasionally reaching fifty per cent. The inferior article is usually whiter and more opaque than the pure. A solution of the pure salt precipitated by muriatic acid and the chloride removed, the solution upon evaporation should leave no residue; if the potassa or soda salt be present, it will be readily discovered in this way.

*Cerates.*—If lard be fused upon poplar buds or gum benzoin, it acquires a very agreeable odor and will keep for a length of time perfectly sweet; it also imparts this keeping quality to ointments and cerates, and is particularly desirable in simple cerate, which should always be free of rancidity when used.

*Dr. Physick's Medicated Ley* for Acid Stomach. ℞—Hickory ashes, 2 oz.; soot, 2 drachms; hot water, a quart. Digest for 24 hours and strain; dose, a wine glass full after eating.

*Hanson's Cholera Morbus Mixture.*—℞—Acet. Morph., 10 grs.; dil. sulph. acid, 25 drops; carb. potass, 2 grains; water, 2 oz.; mix; dose, 25 drops.

*Ansley's Pills.*—℞—Calomel, 2 drachms; comp. extract colocynth, 4 drachms; tartar emetic, 6 grs., powd. ipec. 20 grains; mix, make 108 pills.

*Hill's Bals. Honey.*—℞—Bals. Tolu, 16 parts; storax, 2; opium, 2; honey, 64; alcohol, 260; mix, mascerate 14 days and filter; dose, teaspoonful.

*Blackberry Cordial* for Diarrhœa.—℞—Blackberries, a quart, express the juice, and add water if necessary, to make a pint of juice; add sugar, 1 lb., (avoirdupois,); brandy, 4 oz.; cinnamon, ½ oz.; ginger, ½ oz.; cloves, 1 drachm; heat to boiling point and strain.

*British Oil.*—℞—Barbadoes tar, 1 lb.; oil seneka, 1 gal.; spts. turp., 8 oz;; crude oil amber, a quart; linseed oil, 1½ gal.; bals. sulphur, a quart; mix.

This oil is freely soluble in high-proof alcohol, and conversely the alcohol is soluble in the oil. The mass of the oleaginous washes in vogue now for dressing the hair are simple solutions of castor oil in alcohol or cologne spirit, scented according to the fancy of the perfumer. ℞—Castor oil, 2 ounces ; cologne spirit or alcohol, a pint ; glycerine, a dram ; oil bergamot, a dram—mix.

Olive oil is not soluble in, nor is it a solvent for alcohol, but when equal parts of olive and castor oils are united, the mixture will take up a notable quantity of alcohol, and forms an admirable basis for hair oils. ℞—Castor oil and olive oil, each, ½ pint; alcohol, (95 per ct.,) 2 oz.—mix thoroughly, when settled pour off the turbid oil, filter through very porous thick paper, and perfume to suit the fancy. If olive oil be saponified and the soap decomposed with sulphuric acid, the resulting oleic acid is fully soluble in alcohol, and may be used with castor oil in forming an elegant oleaginous compound for the hair. ℞—Oleic acid, 6 oz. ; castor oil, 6 oz. ; cologne spirit, 4 oz.—mix and perfume to suit. Or, in place of the cologne spirit, use 4 oz. of fine cologne. The oleic acid may be obtained conveniently from castile soap by solution in hot water, and decomposing with sulphuric acid. If the soap be of prime quality the acid will be nearly pure.

Castor oil, when very cheap, may be used as a lamp oil, by adding about 20 per cent of strong alcohol. The oil alone is too thick and viscid, while its high per centage of carbon causes it to smoke ; both these obstacles are gotten over by the use of the alcohol. Query—Would not fish oils of poor quality absorb enough alcohol to overcome their tendency to smoke ?

*Olive Oil*, of the light yellow kind, termed Marseilles, makes a volatile lineament much more satisfactory than can be made from the ordinary sweet oil. The difference in price is little object, when the quality of the two is compared.

*Rhubarb* is one of the few drugs of which it can be said, that the best is the cheapest. The true Turkey is usually so enormously high—a consequence of Russian monopoly—as to preclude all idea of economy in purchasing it. Good samples of China, little inferior to the other, may be had for half the price, or even less, at times.

The solid extract of rhubarb is generally an unsatisfactory preparation. Much of it is no more active than the root itself. The fluid extracts, simple and with senna, are desirable.

Physicians sometimes inadvertently regard the simple and aromatic syrups of rhubarb as essentially the same, with the exception of the little spicing. It should be borne in mind that the simple syrup represents in each teaspoonful say $4\frac{1}{2}$ grains of the root, while the aromatic has only $1\frac{1}{8}$ grains in the like quantity.

A similar error is made with regard to the ammoniacal preparations. In common parlance, Aqua Ammonia is a watery solution, while spirit of ammonia is an alcoholic solution of *caustic* ammonia, and the aromatic spirit is a hydro-alcoholic solution of *carbonate* of ammonia with the aromatic volatile oils. It is by no means a matter of indifference that these several preparations be used indis-discriminately.

*Aloes* is another drug, the quality of which is not indicated by the price. Good Cape aloes is quite equal to the Socotrine variety in efficacy, though much cheaper in price. So, also, the fat varieties of *manna* sold under the term "sorts" are generally more active than the much more costly "large flake."

*Alocin* is attracting some attention in the Northern cities just now; it is highly interesting as a pharmaceutical product, but probably does not merit general adoption in practice.

*Eclectic Remedies.*—Near 4 years ago, Mr. E. S. Wayne, of Cincinnati, examined 18 specimens of these so-called "concentrated" remedies, put up by what is styled the American Chemical Institute of New York. Of the 18, only four proved to be at all what was claimed for them. Their Hyoscyamin was carbonate of magnésia, with tincture of hyoscyamus poured upon it, dried and powdered. Hydrastine contained over 80 per ct. of the same earthy basis. Gelseminum 85 per ct. ditto, and ten others tallied pretty well with these. It is well to bear these things in occasional remembrance, as our profession use this class of remedies sometimes, and the markets are supplied with specimens having this particular brand. Tilden & Co., of New Lebanon, N. Y., manufacture these resinoids, it is believed, of reliable quality.

*Cream Tartar* is greatly adulterated with alum, sulphate of lime, and probably other articles, so much so as to constitute a distinct article of trade under the name "grocers' cream tartar." Sulphate of lime is easily detected by its much more sparing solubility, while alum is recognized by the gelatinous precipitate of alumina upon the addition of ammonia to the solution. Yeast powder is composed of bi-carbonate soda, 1 lb., cream tartar, 2 lbs., rice flour or corn starch, 1 lb., all thoroughly dried and intimately mixed.

*Seidlitz Powders*, as sold in the drug stores, are for the most part quite inefficient, in consequence of the too small quantity of materials used, and also frequently the substitution of Epsom for the more expensive Rochelle salt. A very nice effervescing laxative is put up under the name of Seltzer aperient, which may be successfully imitated in the following combination: ℞—Rochelle salt, 24 drams; bi-carbonate soda, 8 drachms; tartaric acid, 7 drachms; dry the powders thoroughly for two or three days in the hot sunshine, free of dust, mix thoroughly in a warm dry mortar, and put up in dry bottles tightly corked; dose, a tablespoonful, more or less, as indicated. The preparation is conveniently made in the midsummer, and if well put up in wide mouth vials of two or four ounce capacity, keeps good for a twelve month.

*Solution Citrate Magnesia* is quite prone to precipitate the salt in an insoluble state: this may be prevented in a goodly degree by slightly decreasing the amount of magnesia used; and the excess of acid thus obtained, not only aids the solution, but, in our warm climate, renders the preparation more grateful to the patient. The flavoring material obtained by rubbing the lemon upon loaf sugar is more appropriate for this preparation than oil of lemon.

The dry citrate magnesia is very convenient and desirable in country practice—it is usually labeled "soluble," and literally, perhaps, is so; but for practical purposes, it is not. We desire to administer it in cold water, and do not wish to allow the water to become warm in waiting for the solution of the salt; it is therefore best that it be carried in the saddle-bags in a state of fine powder, so that it may be simply stirred up in the water and drank off like cream tartar.

A pleasanter preparation than the Seltzer aperiant may be made in the same manner, substituting 32 drachms of the dry citrate for the 24 of rochelle.

*Sugar-Coated Pills* and granules, prepared by Tilden & Co., for the use of physicians, are now being introduced extensively over the country, while many other parties are catching the idea and offering similar preparations with quite a variation in the range of prices. The writer has used the pills of T. & Co., and found them both convenient and efficient, quite satisfactory in every particular, but how long are they to continue reliable?

Is it probable that the new system of compounding pills and other extemporaneous preparations, upon a huge scale in large manufacturing establishments, will prove beneficial to the profession and the public? There is much room for doubt upon these points, already are some of the evils of the system becoming manifest in the case of Tilden & Co., in the substitution of a pill containing antimony for the true Plummers pill and the invention of an improved compound cathartic pill. How far these substitutions and improvements (?) are to be carried, time will show. A natural strife for the ascendency in the trade will as naturally lead to decline in price and consequent depreciation in quality. The complex character of these preparations renders it exceedingly difficult to determine satisfactorily the quality of any given sample. They must pass through many hands before being administered to the patient—multiplying the dangers of mistaken identity. If mischief be done through incompetency or knavery in the compounds, a wide gap is thrown between the delinquent and the innocent sufferer, forestalling all hope of redress. These and other equally cogent reasons may be assigned for the discouragement of such enterprizes. There is yet another aspect of the case, well worthy of reflection; a strong tendency is becoming more and more manifest towards a *centralization* pharmacy in the large manufacturing establishments; and the conversion of our dispensing shops into mere depots for patent medicines and ready made orthodox prescriptions and compounds—already the conversions of the fluid extracts into infusions, decoctions, syrups and tinctures by mere dilution with the appropriate menstrum is practiced and advocated, soon, it may be, the quality of every preparation we use will be determined solely by the interest of the princely manufacturer or the judgment of his German or Irish "Chemist" away off at the north.

*Compound extract af Colocynth* varies greatly both in price and quality, this is due for the most part to the kinds of aloes and scammony used in its preparation, as also to the fact of the seeds being excluded or not for the colocynth apple, there is an extreme range in cost between virgin scammony and some of the more common sorts.

*Powdered squill* is very difficult to keep in good condition, an excellent plan to avoid loss is to put it up in very small warm dry vials, cork and seal tightly, the vials are opened successively as used and the loss by exposure, if any, will be but trifling.

*Spirit of nitrous ether.*—Several years ago Dr. Squibb, then of the naval laboratory at Brooklyn, examined various samples of this preparation and demonstrated its want of proper efficacy as it is usually found in market. He has long proposed to give us the article manufactured by his improved process, containing the full measure of hyponitrous ether, so far he has not been able to make good his pledge, but is industriouly at work trying to do so.—Poor fellow, in endeavoring to extinguish the flaming ether which resulted in the destruction of his new laboratory at the close of last year, he got his face and hand badly burnt, disfiguring him for life, he is now however hard at work again and will doubtless show us something handsome in the way of pharmaceutical preparations in time.

*Compound Fluid Extract Buchu*—formula for which will be found in Parrish's Practical Pharmacy—has been used freely by the writer and with much satisfaction, it is at least equal to any of the nostrum Buchus' and much better than most of them.

*Diaphoretic mixture*—used after difficult parturition or surgical operation. ℞—Tart. antimony 1 grain, Sulph. Morphine 1½ grain Sp. Nit. ether 2 drachms, camphor water 2 ounces, neutral mixture 4 ounces, mix and direct a tablespoonful every 2 or 3 hours.

*Acetic Syrups.*—Wm. Hodgson Jr., suggests as a remedy for the unstable character of the the syrups of ipecac, senega and squills compound, the reduction of the bulk one eighth by evaporation, making up the measure with acetic acid.

The object aimed at is a good one, and the means employed effectual for the purpose. The property of acetic acid, controlling the crystalization of cane sugar, and its use in making sugar candy, is well known, as also the antiseptic properties of the acid in promoting the decomposition of organic matter. In general terms, the acids are incompatible with tartar emetic, but acetic acid added to the antimonial solution gives no precipitate, and the question whether the presence of this acid will hasten or retard the decomposition of the antimonial by keeping, remains to be determined by experience. So that the propriety of the acetic addition to hive syrup, must remain in doubt until demonstrated satisfactorily by time and observation. Otherwise than as a conservative agent, the acid must be considered objectionable, and the quantity used should be only such as may be required to effect the proposed end. In the case of the ipecac syrup, it is quite probable that one-half or less of the quantity recommended by Hodgson would suffice, and the preparation correspondingly less injured by the addition. We desire occasionally to use syrup of ipecac as an emetic, and much acid would be objectionable in such a case. In the syrup of senega the quantity proposed would generally be less objected to.

*Benzoic Acid*, as it now comes to us, is, for the most part, obtained from the urine of the cow or horse, in which it exists in combination with glycocole, as hippuric acid. It is usually purer and cheaper than the acid obtained from the benzoin by sublimation, and answers every medicinal purpose, though it cannot substitute the latter in perfumery. The two are easily distinguished; the rich and agreeable balsamic odor of the acid from benzoin—due to the contaminating volatile oil—contrasting strongly with the more or less distinctly urinous odor of the other.

*Creosote*, also, is met with now from two distinct sources, namely: wood and coal tar. They differ considerably, and may be readily distinguished by comparison. The coal tar creosote is the cheapest, and it does not appear that it is inferior in efficacy to the old article.

